# Pediatric rehabilitation delivery: discussion is an antidote to disconnection and discontent

**DOI:** 10.1186/s13584-024-00619-7

**Published:** 2024-08-06

**Authors:** Maurit Beeri

**Affiliations:** ALYN Hospital Pediatric and Adolescent Rehabilitation Center, Jerusalem, Israel

**Keywords:** Pediatric rehabilitation, Rehabilitation day care center, Complex medical needs, Policy, physiotherapy

## Abstract

Pediatric rehabilitation is fundamentally different from that of adults. Child physiology differs significantly from that of adults, necessitating specialized rehabilitation approaches. Unique injuries and varying metabolic rates underscore the need for tailored care, which changes over the years as the child grows and develops. Waiserberg’s paper, “When Everyone is Responsible, No One Takes Responsibility”: Exploring Pediatric Physiotherapy Services in Israel,” sheds light on a critical issue. While senior practitioners oversee policy implementation and service delivery, practical physiotherapy treatment lacks continuous monitoring. This is a critical issue. Ideally, every child who requires long-term clinical therapeutic interventions to keep up with peers in mobility, communication and cognitive skills should be assessed by specialists several times throughout the school years, and their personalized rehabilitation plan discussed, reviewed, and adjusted according to their progress. The absence of a standardized protocol for overseeing and directing comprehensive rehabilitation plans leaves therapists feeling alone and adrift, whether working in schools or medical settings. Such an assessment would be an opportunity to create a registry, which is currently nonexistent. The collected data would be a priceless resource in policy decision-making and service planning.

Child physiology is different from that of adults, necessitating specialized rehabilitation approaches. The very need for rehabilitation is often the result of injuries and diseases which are unique to children. Children have the ability to survive conditions which would be fatal in adults. The young body’s metabolism and recuperation mechanisms are not the same as the adult’s [1]. Not only is the array of injuries and types of diseases of importance in the rehabilitation process, but the plasticity of the young brain and the recovery from neuronal disruption make children do better in the long term in some types of damage while being more susceptible to worse outcomes in others [2]. These differences explain why pediatric rehabilitation requires an understanding and expertise of its own.

A cardinal feature of pediatric rehabilitation is that treatment goals continuously change. Typically, developing children achieve different skills and behaviors as they grow. A child of 18 months is not expected to jump or to communicate in complex syntax. However, if these milestones have not been attained by four years of age, this demonstrates a significant developmental delay. Therefore, pediatric therapists must always look to the horizon and beyond. Every step forward is but a gateway to new challenges. Thus, the implementation of the ubiquitously- embraced International Classification of Functioning, Disability and Health framework (ICF) [3] is very different from the way it is used in adults. In adult rehabilitation, goals are defined as end-points in regaining lost or damaged functional abilities. Pediatric rehabilitation, on the other hand, focuses not only on acquiring the re-use of previously achieved developmental milestones and compensating for permanent loss of function due to impairment, but also on the acquisition of ongoing new age-appropriate skills as the child grows. In 2012, Peter Rosenbaum suggested a child-appropriate ICF - equivalent terminology, in which “Activity” is defined as “Function”, “Environment” equals “Family”, Body structure and function are expressed as “Fitness” and “Personal factors” as “Fun”. A is frequently added to Rosenbaum’s seminal five [4]: Future. The 6th element is why pediatric rehabilitation will always require long term programs. Always looking towards the future requires periodic re-visiting of treatment goals. The child, family and the therapists (physical therapists, occupational therapists, speech language pathologists, educators, psychologists and more) should come together to discuss preferred treatment options and decide which goals should take precedent at any given point during the child’s formative years. The unique approach to children and a useful format of assessing the overall plan should be adjusted to the entire daily life of a child.

Waiserberg’s paper “When Everyone is Responsible, No One Takes Responsibility”: Exploring Pediatric Physiotherapy Services in Israel” (REF) addresses the problematic situation in which the interviewees, amongst whom are some of the most senior practitioners in charge of policy implementation and service delivery in Israel, feel that provision of physiotherapy is not adequately monitored nor continuously assessed. Waiserberg’s paper highlights important and challenging issues in service delivery. It does not address another important issue, the lack of a standard protocol for oversight and monitoring the direction of the entire rehabilitation plan. I would argue that the way children receive treatment throughout their formative years, with no one responsible for the entirety of a long-term program, is of deep concern. Without such a tool, it is little wonder that therapists, whether providing services at school or in a medical setting, feel adrift.

Ideally, every child who requires long term clinical therapeutic interventions to keep up with peers in mobility, communication and cognitive skills should be assessed by specialists several times throughout the school years, and their personalized rehabilitation plan reviewed and adjusted according to their progress [5]. Such assessment would enable oversight, detect flaws in provision, and direct therapists [6]. It would be an opportunity to re-assess the need for assistive devices, highlight socio-economic risk factors and to identify potential medical issues that may require investigation and intervention (e.g. neurological, orthopedic and nutritional).

These assessments can be provided through the HMO’s or outsourced to the specialized multi-disciplinary clinics operating in pediatric rehabilitation centers. The child’s community therapists should be a part of the assessment whenever possible, provide data and participate in deciding the recommendations, in order to prevent a gap between what has been planned and the actual implementation.

Coordinating between separate government services (e.g. health and education) may seem an insurmountable task. However we can look at the success of a third pediatric rehabilitation provider in Israel, not assessed in Weisberg’s paper. The Rehabilitation Day Care (RDC) system, operating since the year 2000 under a designated law [7], provides services for children ages 6 months to three years, and is overseen by both the Ministry of Health and the Ministry of Welfare Services. Within this framework, ongoing data regarding the provision of therapies is collected and analyzed [8, 9]. Teams work together to discuss and deliver a unified plan. The joint oversight by the health and welfare ministries could be a model for bridging the gap between Health and Education ministries. The Rehabilitation Day Care system has its faults, too. Provision of therapies in the RDC’s is dictated by the law and may not tailor the dosage or blend of therapies to the child’s condition, and does not allow tailoring the program to shift emphasis or adapt it to the child’s health condition or developmental pace.

When Waiserberg describes physical therapists who feel that “they operate alone” within a system that does not recognize or acknowledge their contribution and expertise, it is a stark rebuke that highlights the lack of teamwork. Ideally, educators see the therapists as a useful resource that not only provide out-of-class therapies for a particular child, but assist in implementing goal attainment and help the carry-over of in-session achievements, practice and progress throughout their daily routine. One way of promoting such understanding is through educating teachers in promoting the knowledge of what therapy is, and how to address a child with a particular challenge. Courses in the approach to children with cerebral palsy, traumatic brain injury and feeding issues, for instance, have recently been added to the Ministry of Education’s Continuing Education platforms.

According to Waiserberg’s findings, senior therapists feel that “Physiotherapy is secondary in the education system, even within the health professions…” I suspect that other therapists working in an environment lacking a supportive coordinated team approach would probably express similar misgivings. Providing a coordinated framework with scheduled team meetings where discussion is encouraged would be the obvious way of addressing such issues. Each child should have a designated multidisciplinary “pod” of service providers, across the various modalities and regardless of their affiliation, (school/ HMO/ family initiated), meeting periodically to discuss the program. These do not have to be face-to-face meetings (remote team-building is as feasible nowadays as is remote therapy). When all stakeholders charged with the rehabilitation input in a child’s daily routine plan a comprehensive program as a team of equals, none of them should feel alienated and disrespected.

A coordinated planned model will also probably result in better long-term results for the child, though that still needs to be proved with proper data. Such an assessment would be an opportunity to create a registry, which is currently nonexistent. The collected data would be a priceless resource in policy decision making and service planning.

There is one major obstacle to the planned program model, and it is the dearth of medical oversight available by specialists. There are few pediatric physiatrists in Israel, and most work within the medical centers and provide short –term rehabilitation care. Therefore, periodic assessment in the multidisciplinary outpatient clinics has to be coordinated with the community service providers.

## Conclusions

I congratulate Waiserberg for bravely identifying the plight of the lonely therapist adrift in a sea of disconnected systems and discontented professionals. Her call for policymakers to engage in coordination and cooperation as the key to promoting better service delivery is the right call.Having a standardized protocol for oversight that defines a “group leader” for each child who can review progress on the overall plan for the child’s rehabilitation and development and coordinate the various therapists and others, agencies (medical and school), in medical and community settings, and help ensure optimal outcomes.

It is by making the stand-alone islands of care into an allied land of opportunities that we can work towards a brighter horizon for both children and professionals.

## Data Availability

Data sharing not applicable to this article as no datasets were generated or analysed during the current study.

## Abbreviations

RDC: Rehabilitation Day Care
ICF: International Classification of Functioning, Disability and Health framework
